# Evolution of the Small Family of Alternative Splicing Modulators Nuclear Speckle RNA-Binding Proteins in Plants

**DOI:** 10.3390/genes11020207

**Published:** 2020-02-18

**Authors:** Leandro Lucero, Jeremie Bazin, Johan Rodriguez Melo, Fernando Ibañez, Martín D. Crespi, Federico Ariel

**Affiliations:** 1 Instituto de Agrobiotecnología del Litoral, Universidad Nacional del Litoral, CONICET, FBCB, Centro Científico Tecnológico CONICET Santa Fe, Colectora Ruta Nacional No 168 km. 0, Paraje El Pozo, Santa Fe 3000, Argentina; lucero@santafe-conicet.gov.ar; 2 CNRS, INRA, Institute of Plant Sciences Paris-Saclay IPS2, Universite Paris Sud, Universite Evry, Universite Paris-Diderot, Sorbonne Paris-Cite, Universite Paris-Saclay, 91405 Orsay, France; jeremie.bazin@ips2.universite-paris-saclay.fr; 3 Instituto de Investigaciones Agrobiotecnológicas, CONICET, Universidad Nacional de Río Cuarto, Río Cuarto 5800, Argentina; jsrodriguezm_1@uqvirtual.edu.co (J.R.M.);

**Keywords:** lncRNA *ENOD40*, RBP1, nuclear speckle RNA-binding proteins, symbiotic nodule development, alternative splicing, evolution, legumes

## Abstract

RNA-Binding Protein 1 (RBP1) was first identified as a protein partner of the long noncoding RNA (lncRNA) *ENOD40* in *Medicago truncatula*, involved in symbiotic nodule development. RBP1 is localized in nuclear speckles and can be relocalized to the cytoplasm by the interaction with *ENOD40*. The two closest homologs to RBP1 in *Arabidopsis thaliana* were called Nuclear Speckle RNA-binding proteins (NSRs) and characterized as alternative splicing modulators of specific mRNAs. They can recognize in vivo the lncRNA *ALTERNATIVE SPLICING COMPETITOR* (*ASCO*) among other lncRNAs, regulating lateral root formation. Here, we performed a phylogenetic analysis of NSR/RBP proteins tracking the roots of the family to the Embryophytes. Strikingly, eudicots faced a reductive trend of NSR/RBP proteins in comparison with other groups of flowering plants. In *Medicago truncatula* and *Lotus japonicus*, their expression profile during nodulation and in specific regions of the symbiotic nodule was compared to that of the lncRNA *ENOD40*, as well as to changes in alternative splicing. This hinted at distinct and specific roles of each member during nodulation, likely modulating the population of alternatively spliced transcripts. Our results establish the basis to guide future exploration of NSR/RBP function in alternative splicing regulation in different developmental contexts along the plant lineage.

## 1. Introduction

RNA-binding proteins (RBPs) participate in the control of gene expression at every step after transcription, including Alternative Splicing (AS), silencing, RNA decay, and translational control [1]. In *Arabidopsis thaliana*, several hundreds of proteins have been predicted to bind RNAs [2]. However, only a small subset of RBPs has been functionally characterized in plants. The nuclear speckle RNA binding proteins (NSRs) are a family of RBPs that act as regulators of AS and auxin-regulated developmental processes such as lateral root formation in *Arabidopsis thaliana* [3]. These proteins were shown to interact with some of their alternatively spliced mRNA targets and with a structured noncoding transcript named *ALTERNATIVE SPLICING COMPETITOR* (*ASCO*) lncRNA [3]. Overexpression of *ASCO* was shown to affect AS of a subset of mRNA regulated by NSRs, in a similar manner as in *nsra/b* double mutants, and *ASCO* was also shown to compete in vitro with the binding of one AS mRNA target. These observations led to proposing that plant lncRNAs are able to modulate AS by hijacking RBPs such as NSRs [4]. More recently, a genome-wide analysis was performed to monitor the global role of NSRs in RNA processing and AS, as well as the direct interacting mRNAs of NSRs [5]. This approach served to assign a new role for NSRs in the control of flowering time regulators, as well as to suggest that NSRs participate in the crosstalk between auxin and the immune response pathway. Interestingly, a subset of lncRNA, in addition to *ASCO*, was found to interact directly with NSRs. In the model legume *Medicago truncatula*, NSRs’ closest homolog, RNA-BINDING PROTEIN 1 (RBP1), here renamed MtNSR1, was localized in nuclear speckles where many components of the splicing machinery accumulate in plant cells. Remarkably, MtNSR1 also interacts with a highly structured lncRNA, *EARLY NODULIN40* (*ENOD40*), which participates in root symbiotic nodule organogenesis [6,7]. *ENOD40* was found both in the nucleus and the cytoplasm, and it is able to relocalize MtNSR1 from nuclear speckles into cytoplasmic granules during nodulation. These observations hint at a role of the lncRNA *ENOD40* in nucleocytoplasmic trafficking of MtNSR1 [8]. The identification of *NSR-ASCO* interaction in Arabidopsis and *MtNSR1-ENOD40* in Medicago suggests the existence of conserved RBP-lncRNA modules controlling AS across species. In this work, we explore the phylogenetic history of the NSR family of RBPs, and we identify the acquisition of particular protein domains throughout evolution, likely affecting their sub-cellular localization and function. Furthermore, we analyzed the expression of each *NSR* gene throughout nodule development, both in *Medicago truncatula* and *Lotus japonicus*, and their co-expression with *ENOD40* lncRNAs, as well as its correlation with AS changes induced during these processes. Distinct expression profiles of NSRs hint at specific roles during nodulation.

## 2. Materials and Methods

### 2.1. Identification of NSR in the Plant Kingdom, Phylogenetic Analysis, and Conserved Protein Motif Characterization

NSRa (AT1G76940) and NSRb (AT1G21320) protein sequences from *A. thaliana* were used as the query to perform BLASTP searches against the Phytozome database [9] in Chlorophyte and Embriophyte, using default parameters. For legumes, we also used the Legume Information System [10] database to retrieve data.

Multiple sequence alignments were performed using Muscle [11]. Mesquite v3.51 (http://www.mesquiteproject.org) [12] was used to build all the matrices for phylogenetic analysis. In order to obtain the trees of NSR proteins, the maximum likelihood optimality criterion was implemented using RAxML v.8 [13], with the GTR model. Branch support was estimated with the rapid bootstrap method as implemented in RAxML v.8, with 100 replicates. The final trees were drawn with Figtree v1.4.4 [14].

To identify conserved motifs in NSR proteins across the Embriophytes, MEME v.5.0.4 [15] was used with default settings, with the motif width set to ≥10 and ≤150 and a maximum number of motifs to find set to 20.

### 2.2. RNA Expression Analysis of NSR Genes in M. Truncatula and L. Japonicus Nodules

Data from [16] were directly extracted from the authors’ website. Available RNA-seq data were also used to characterize the expression of the two *NSR* genes found in *M. truncatula* and *L. japonicus*, respectively. For *M. truncatula*, we used the dataset published by [17]. Root samples were harvested at 0.5, 1, 3, 6, 12, 24, 36, and 48 h post infection (hpi) with *Sinorhizobium meliloti*. For *L. japonicus*, we used the dataset published by [18]. Root hairs from inoculated vs. non-inoculated roots were harvested 72 hpi. Roots were also compared to nodule primordia (7 days post infection, dpi) or mature nodule (24 dpi). Raw RNAseq datasets were downloaded from European Nucleotide Archive [19] with the following accession numbers: PRJNA269201 and PRJNA422278. Sequencing adaptors were trimmed using Cutadapt (Martin, 2011). Medicago and Lotus transcript abundance was quantified using a pseudo alignment read count method with kallisto [20], on all transcripts of the *Medicago* genome annotation v5 (https://medicago.toulouse.inra.fr/MtrunA17r5.0-ANR/) [21] and the *Lotus* genome annotation v3 [22], respectively. Heatmaps were generated with the pheatmap R package using the Transcript Per Million reads (TPM) values generated by kallisto. AS analysis was performed using SUPPA [23] and TPM values. The AS event types considered by SUPPA were: Exon Skipping (ES), Alternative 5′ (A5S) and 3′ splice sites (A5S), Mutually exclusive Exons (MX), and Intron Retention (IR). For each event, SUPPA calculated the inclusion parameter Ψ, which was defined as the ratio of the abundance of transcripts that included one form of the event over the abundance of the transcripts that contained either form of the event. Statistical analysis of differential AS events was performed with SUPPA using the empirical method, and only events with *p* < 0.01 and |ΔΨ| > 0.1 were retained as significant.

## 3. Results

### 3.1. The NSR Protein Family Predates the Origin of Vascular Plants

In order to explore the evolutionary relationships of NSR proteins in plants, inferred through phylogenetic analysis, we first used *Arabidopsis thaliana* NSRa and NSRb protein sequences as queries to perform BLASTP searches against Chlorophyte and Embryophyte available genomes (Supplementary Table S1). Notably, no hits were obtained in the available Chlorophyte genomes. Significant hits were only found in land plants in Embryophytes. Interestingly, no hits were obtained in the basal Embriophyte species *Physcomitrella patens* and *Sphagnum fallax*, but four NSRs genes were identified in the genome of the Bryophyte (moss) *Marchantia polymorpha* (Supplementary Table S1) and three in the genome of the basal Tracheophyte *Selaginella moellendorffii*. At least one NSR protein was identified in all plant species analyzed in Bryophytes. The phylogenetic tree of NSR proteins resulted in being almost fully resolved with most internal branches well supported. Three main clades appeared in accordance to the phylogeny of land plants. Clade 1 included proteins of basal Tracheophyte, Bryophyte, and *Amborella*. All the monocot members identified were included in clade 2, along with two sequences of *Amborella* and one of *M. polymorpha*. Finally, clade 3 grouped all eudicot NSRs (Supplementary Figure S1). The NSRs proteins from the most basal extant angiosperm *Amborella trichopoda* were closely related to the *S. moellendorffii* and *M. polymorpha* sequences in clade 1, as expected. On the contrary, NSR proteins from the monocot species included in the analysis, restricted to Poaceae (*Oryza sativa*, *Brachypodium distachyon*, *Setaria viridis*, and *Zea mays*), grouped together in clade 2. Within the Poaceae family, the number of *NSR* members was greater in *Z. mays*, compared with its close relatives *S. viridis* and *O. sativa*. Interestingly, during evolution of flowering plants, the number of *NSR* genes was reduced, as shown by most of the eudicot species incorporated to our study grouped in clade 3, which only had two *NSR*-coding genes or even just one *NSR* as in the *Capsella* genus (Supplementary Figure S1 and Table S1).

**Figure 1:**
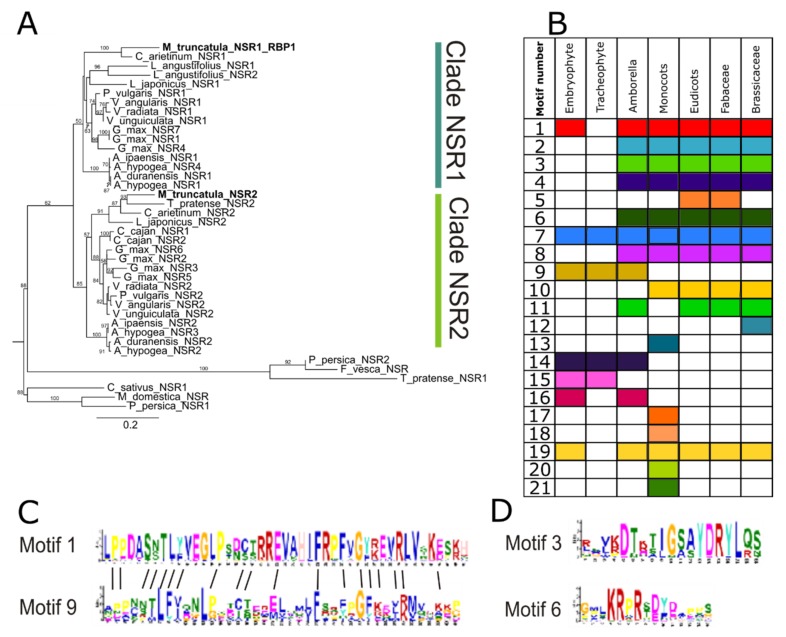
**Nuclear Speckle RNA-binding proteins** NSR phylogeny of selected eudicot families and motif analysis of NSR proteins in vascular plants. (**A**) Maximum likelihood tree of NSR proteins in Fabaceae; *M. truncatula* sequences are bolded. Bootstrap values are indicated above branches. (**B**) Protein motifs’ occurrence in land plants. The 21 conserved motifs identified with MEME are detailed in Supplementary Figure S3. (**C**) Comparison of motifs 1 and 9 depicting conserved amino acid residues shared between both motifs (see the text for more explanation). (**D**) Weblogo of motifs 3 and 6 exclusive to flowering plants. The Nuclear Localization Signaling (NLS) can be visualized in position four to seven of motif 6.

Considering that the first characterized NSR protein belonged to *Medicago truncatula* (MtRBP1/MtNSR1) [8], we further focused our study on NSR proteins in the Fabaceae family (legumes). To this end, we identified the NSR protein sequences from 14 species, based on public repositories. As expected, MtNSR1 was recovered by the BLASTP search in *M. truncatula,* using NSRa or NSRb from *A. thaliana* as query entries. Interestingly, an additional uncharacterized NSR coding gene was identified in *M. truncatula*, hereafter called *MtNSR2*. A phylogenetic analysis was performed using NSR sequences from *Cucumis sativus* (order Cucurbitales) and *Prunus persica* and *Fragaria vesca* (order Rosales) as the outgroup (Supplementary Table S2). Two supported clades diverged early in the history of legumes, here denominated the NSR1 clade (due to the presence of MtNSR1) and the NSR2 clade, respectively (Figure 1A). Notably, all the species included had at least two *NSR*-coding genes, and all the NSR proteins from the Fabaceae family resulted in a monophyletic clade, except from *T. pratense* NSR2, which seemed more closely related to the outgroup proteins. A possible phylogenetic artifact may explain this observation about *T. pratense* NSR2. Long Branch Attraction (LBA) is a recurrent source of error in phylogenetic inference [24], when branches with a high substitution rate appear as closely related to other branches, which in fact do not share a common evolutionary history. This may probably be the case for *T. pratense* NSR2. The advance in genome sequencing of more legume species in the next few years will aid to shed light on this matter.

Additional lineage-specific gene duplications have occurred in *Arachis hypogaea* and *Glycine max*, with four and seven *NSR* genes, respectively. Notably, *Lupinus angustifolius* has two *NSR* genes, but instead of having one member from each NSR clade, both of their protein sequences cluster together in the NSR1 clade, hinting at a recent duplication, whereas the NSR2 copy appears to be lost in this species. A similar configuration was observed for *Cajanus cajan*. However, while in *L. angustifolius*, both proteins belong to the NSR1 clade, *C. cajan* NSRs are included in the NSR2 clade of legumes.

### 3.2. Changes in Protein Domain Composition Suggest Distinct Roles for NSRs in Different Plant Lineages

To further evaluate the structural diversity of NSR proteins, we examined the presence of conserved motifs in the sequences belonging to land plants. Three conserved domains were previously identified between *Medicago truncatula* MtNSR1 and proteins from other eudicots [8]: (i) a nuclear localization signaling motif; (ii) a conserved motif of unknown function; and (iii) the C-terminal RNA Recognition Motif (RRM) domain (Supplementary Figure S2). Here, we considered all protein sequences used to build the full phylogenetic tree (Supplementary Figure S1) to search novel conserved motifs. Using MEME [15], 21 conserved motifs were identified across the NSR proteins evaluated (Figure 1B; Supplementary Figure S3). We found three motifs present across all plant species: motifs 1, 7, and 19. Strikingly, the C-terminal RRM domain previously characterized in MtNSR1 (Supplementary Figure S2) here resulted in being partially associated with motif 1 and motif 2, with 45 and 57 residues, respectively. Motif 1 is the most represented domain in NSR proteins, only absent in Selaginella, and motif 2 is present in monocots and eudicots, but surprisingly not in the basal angiosperm Amborella. Although motif 1 is not present in Selaginella NSRs, a close examination of motif 9 and motif 1 suggested that they were highly similar, where their most represented residues were present in both motifs, respectively (Figure 1C). Taking into account that motif 1 and 9 were present in Marchantia NSRs, we proposed that at least part of the C-terminal RRM domain predated the origin of land plants, whereas it was completed as the so-called motif 2 after the origin of flowering plants before the split of monocots and eudicots.

The other two motifs previously identified in MtNSR1 (Supplementary Figure S2; [8] were now found to be exclusive to angiosperms, denominated motif 3 and motif 6 (Figure 1E; Supplementary Figure S3). In particular, we observed that the KRPR Nuclear Localization Signaling (NLS) domain resulted in being included in motif 6 (Figure 1D; Supplementary Figure S3). The results of the analysis performed by MEME indicated that there were some lineage-specific motifs, such as motifs 13 and 14 in *Z. mays*. Others were unique to some plant families, like motifs 13, 17, and 18, or motif 12, from Poaceae and Brassicaceae, respectively (Figure 1B; Supplementary Figure S3). Altogether, our results indicated that the small family of NSR proteins was characterized by several conserved motifs hinting at similar molecular roles. However, the loss and gain of particular motifs might have substantially modified the function of NSRs during the evolution of plants. For instance, motif 6, which contains the canonical NLS, appeared late in the evolution of land plants, indicating that NSR proteins could enter the cell nuclei since the appearance of the angiosperms. According to this hypothesis, sub-cellular localization of Embryophyte and Tracheophyte NSR may likely be essentially cytoplasmic, likely hindering their eventual participation in nuclear-localized alternative splicing. However, we could not exclude that Marchantia or Selaginella NSR proteins enter the nuclei through an alternative mechanism. If so, then these basal plant groups should require different molecular mechanisms to transport NSR proteins to the nucleus and to incorporate them into speckles, as reported in Arabidopsis [3] and Medicago [8]. Additionally, we found that the conserved motif of unknown function previously found in eudicot species [8], here included in motif 3, was also restricted to flowering plants, further hinting at domain gaining throughout evolution. Altogether, our results suggested that the molecular function of NSR experienced a major shift in the evolution of land plants, including changes in its subcellular localization, and that the ability to modulate alternative splicing might have been acquired within angiosperms.

### 3.3. Alternatively Spliced Variants of NSR Genes May Compensate Lack of Gene Family Expansion

**Figure 2:**
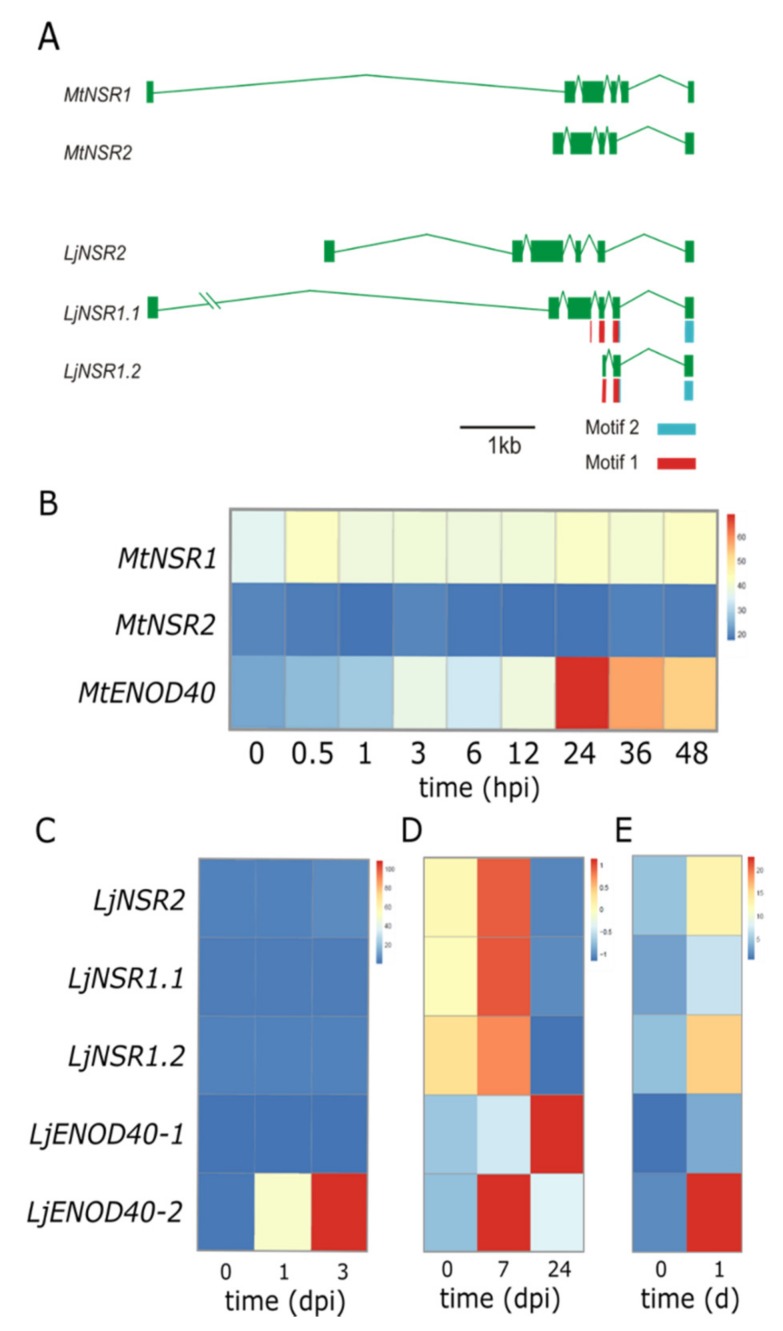
Gene structure and alternatively spliced variants of NSR genes and their expression profile, together with the lncRNAs ENOD40 of *Medicago truncatula* and *Lotus japonicus* during nodulation. (**A**) Exon-intron structure of *NSRs* genes in both species. The alternative splicing variant LjNSR2.2 includes part of exon 4 and exons 5 and 6, which coded for motif 1 (blue box) and motif 2 (orange box). (**B**) Heat map of *NSRs* and *ENOD40* expression in inoculated roots of *M. truncatula* [17]. (**C**) Heat map of *L. japonicus NSR1.1*, *NSR1.2*, *NSR2*, *ENOD40-1*, and *ENOD40-2* expression in root hairs post inoculation with rhizobia, (**D**) in a time course of nodule formation (7 dpi = nodule primordia, 24 dpi = fixing nodule), and (**E**) in response to exogenous treatment with Nod factors [18]. For C to E, the Heatmap shows the mean of normalized expression (Transcript Per Million reads (TPM)) for all replicates for each time point. dpi stands for days post inoculation. hpi stands for hours post inoculation.

The number of exons of NSR genes showed a remarkable conservation in the eudicot species examined. As previously described for *A. thaliana* from the Brassicaceae family [3], the legume species *M. truncatula* and *L. japonicus* also had between five and six exons (Figure 2A). Interestingly, *L. japonicus NSR1* presented two alternative mRNA isoforms, *LjNSR1.1* and *LjNSR1.2*. In the latter, exons 1, 2, 3, and part of exon 4 were lost, whereas exons 5 and 6 were retained (Figure 2A). This alternatively spliced variant was translated into a peptide that retained the complete coding region for motif 2 (exon 5 and 6) and part of motif 1 (exons 3, 4, and 5), but had lost all the other conserved motifs of the legume family, including the NLS located in motif 6. Interestingly, the C-terminal RRM domain responsible for RNA binding was included in motifs 1 and 2, which were present in *LjNSR1.2*. Therefore, although eudicots experienced a reduction of NSR coding genes, and novel variants of NSR proteins emerged as a result of the alternative splicing suffered by primary *NSR* RNAs. The dramatic change in the domains’ compositions between LjNSR1.1 and LjNSR1.2 indicated that they may exert different roles in RNA metabolism.

### 3.4. Transcriptional Behavior of NSR Genes in Legumes Hints at Distinct Roles During Nodulation

In *Arabidopsis thaliana*, only *NSRb* is transcriptionally responsive to exogenous auxin treatment, and no phenotype was described for *nsra* or *b* single mutants in response to auxin, in contrast to the double mutant *nsra/b* [3]. However, *nsra* single knockout plants exhibit an early flowering phenotype in contrast to *nsrb* single mutants, due to the indirect regulation of the *FLC* locus by NSRa [5]. These findings hint to overlapping or distinct roles of NSR proteins depending on the developmental context in which they participate.

As previously mentioned, the sole NSR characterized so far apart from AtNSRs belongs to the model legume *Medicago truncatula* (MtNSR1) [8]. Legumes have the remarkable property of forming an endosymbiotic interaction with a group of bacteria collectively referred to as “rhizobia” [25]. Symbiotic development culminates in the formation on roots of a new organ called the root nodule, which is colonized by rhizobia that fix atmospheric dinitrogen to ammonia, to the benefit of the host plant.

We showed above that in *M. truncatula*, an additional NSR gene exists, *MtNSR2*. Similarly, two *NSR* genes were identified in the model legume *L. japonicus*. Thus, we wondered how the two *NSR* genes behave throughout nodulation in both model legumes *M. truncatula* and *L. japonicus*, for which high quality publicly available transcriptomic datasets are available [17,18]. Based on these transcriptomic approaches, we assessed the behavior of each gene in response to symbiotic bacteria or the rhizobial-secreted lipochitooligosaccharidic molecules called Nod factors. Furthermore, we compared the transcriptional response of *NSR* genes with the behavior of the lncRNAs *ENOD40* (in the case of *L. japonicus*, the two *ENOD40* existing genes), given the interaction shown in *M. truncatula* and the capacity of *MtENOD40* to modulate the sub-cellular localization of MtNSR1 [8]. According to the time course of *M. truncatula* roots inoculated with rhizobia, *NSR1* transcript levels were steadily more abundant than *NSR2* between 0 and 48 h post inoculation (hpi). The early nodulin transcript *ENOD40*, however, dramatically peaked at 24hpi (Figure 2B). In *L. japonicus*, *NSR* genes did not respond in root hairs to rhizobia inoculation, whereas *LjENOD4-2* was strongly induced at three days post inoculation (dpi; Figure 2C). Interestingly, comparing transcript levels of Lotus *NSRs* and *ENOD40s* later in nodule development, the two isoforms of *NSR1*, *NSR2*, and *ENOD40-2*, they all peaked in nodule primordia at 7 dpi and decreased at 24 dpi, when *ENOD40-1* peaked (Figure 2D). Furthermore, it is worth noting that in response to nod factors (24 h treatment), only *ENOD40-2*, *NSR1.2*, and *NSR2* were induced, whereas *ENOD40-1* and *NSR1.1* both remained at low levels (Figure 2E).

**Figure 3:**
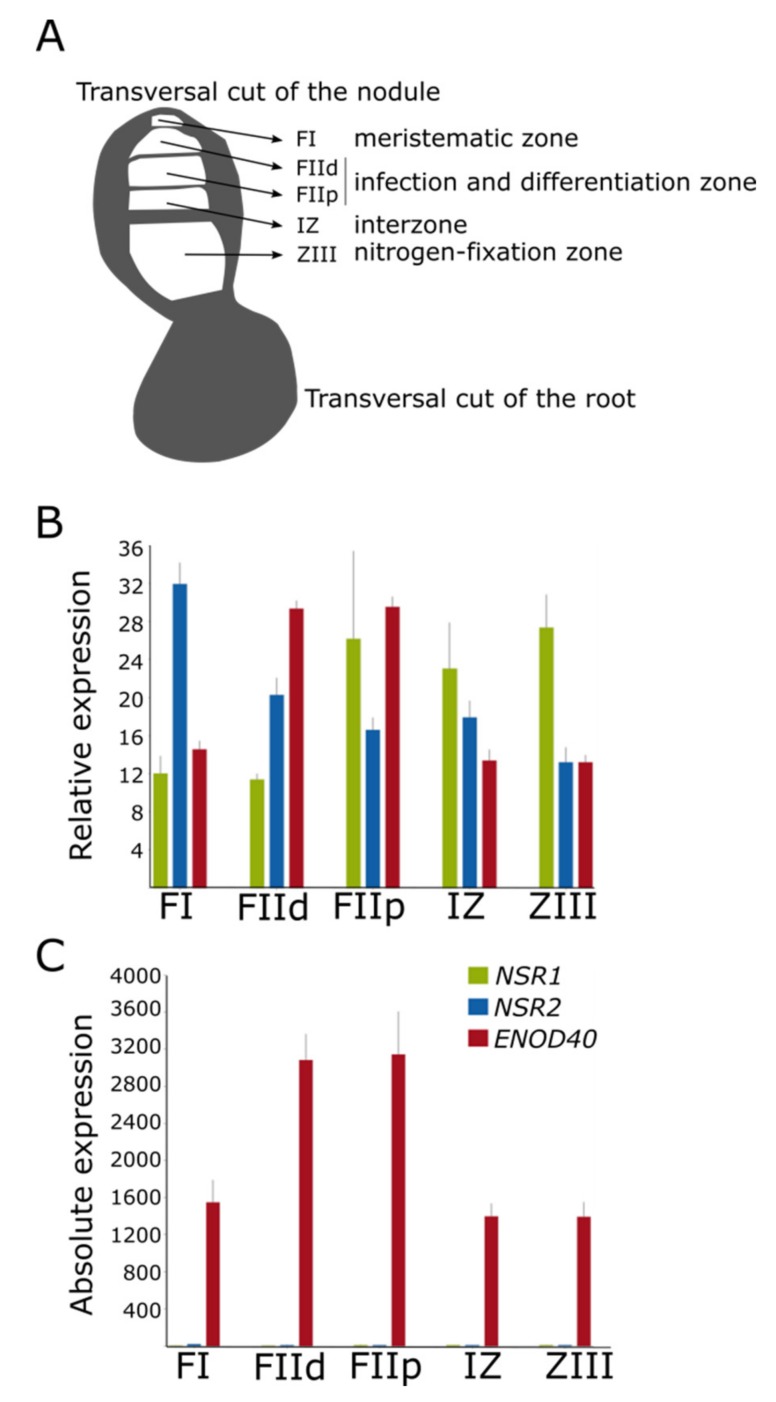
Expression pattern of NSR genes and the lncRNA ENOD40 in each zone of the *Medicago truncatula* mature nodule, based on [16]. (**A**) Schematic representation of a transversal cut of a *M. truncatula* root and mature nodule, indicating the zones isolated by laser micro-dissection. Fraction I (FI) corresponds to the nodule meristematic zone; the zones below FI correspond to samples collected as a distal and a proximal Fraction (FIId and FIIp, respectively) and corresponding to cells undergoing differentiation or infection; the Interzone (IZ) separates the fractions above from the nitrogen-fixation zone ZIII. (**B**) Relative transcript levels of NSR1, NSR2, and ENOD40 in each zone of the mature nodule. (**C**) Absolute levels of the same genes shown in **B**.

In order to further characterize *NSR* and *ENOD40* expression during nodulation, we analyzed the laser micro-dissection RNA-Seq dataset (Figure 3A; [16]). We determined in which regions of the *Medicago* nodule each gene transcript was accumulated. Strikingly, although *NSR1* and *NSR2* RNA levels remained constant during nodulation (Figure 2B), their distribution along the mature nodule showed different patterns (Figure 3B). *NSR1* accumulation was lower in meristematic and infection zones and relatively higher in zones of differentiation and nitrogen fixation. On the other hand, *NSR2* transcript levels were higher in the meristematic zone than in the rest of the nodule, where levels remained low and steady. *ENOD40* transcriptional accumulation was remarkably high throughout the whole nodule (Figure 3C) and relatively higher in zones of infection and differentiation. Altogether, our analyses indicated that dynamic interaction in different cell types occurring between NSRs and *ENOD40* alternative NSR-*ENOD40* modules may take place during nodulation, having a tightly regulated impact on the alternatively spliced population of mRNAs in symbiosis.

### 3.5. Distinctive Populations of Alternatively Spliced mRNAs Characterize the Progression of Nodule Development

**Figure 4:**
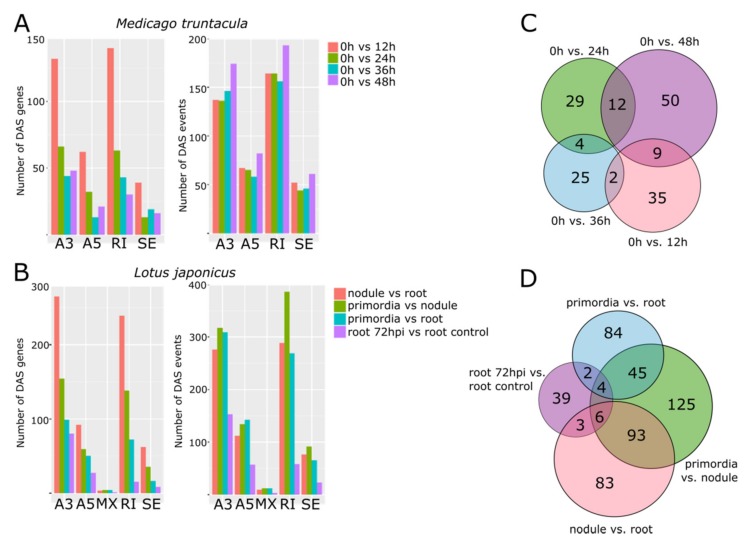
Alternatively processed mRNAs profile during nodule development in *M. truncatula* and *L. japonicus*. Alternative isoforms between samples were identified using SUPPA. The number of genes or events (indistinguishable from what gene) suffering Differential Alternative Splicing (DAS) were scored in (**A**) *M. truncatula* and (**B**) *L. japonicus*. A3 and A5 stand for Alternative processing of the 5′ and 3′ ends, respectively; IR stands for Intron Retention; MX stands for Mutually exclusive Exons, and ES stands for Exon Skipping. The identity of the DAS genes was compared between samples showing little overlapping in (**C**) *M. truncatula* and (**D**) *L. japonicus*.

In *A. thaliana*, the NSRa/b-*ASCO* module regulates the AS output of a subset of NSR target mRNAs, notably several related to auxin responses [3]. Considering the differential transcriptional behavior of *NSR* genes and *ENOD40s* during nodulation, both in *M. truncatula* and *L. japonicus*, we wondered how this correlates with the dynamic population of alternatively spliced mRNAs in this process. Hence, we quantified the number of AS events and alternatively spliced genes comparing each condition using SUPPA (Figure 4, see Materials and Methods, for the full list of genes in both species; see Supplementary Table S3). To this end, we classified the alternatively RNA splicing events into alternative 3′ or 5′ ends, intron retention or exon skipping. Remarkably, the biggest impact on the AS population resulted when comparing non-inoculated roots with nodules in *L. japonicus* even with the shortest time point post inoculation assessed, e.g., 12 hpi, in *M. truncatula*. In both cases, a prominent change was observed for genes alternatively spliced at their 3′ end, as well as those showing intron retention. Overall, the differentially spliced events, independently of the number of genes, remained homogeneous across inoculated samples compared to non-inoculated roots; alternatively spliced 3′ ends and intron retention being the two more highly scored events (Figure 4A,B).

Strikingly, when comparing the identity of the alternatively spliced genes in each time point, most of them turned out to be unique to each case, both in *M. truncatula* and *L. japonicus* (Figure 4C,D). A progression of AS was observed along the time points of rhizobial infection (roots, vs. nodule primordia vs. mature nodule), suggesting a dynamic regulation of splicing during the establishment of this organ in both species. The highest number of differential genes in *L. japonicus* was observed between the mature nodule and the nodule primordia (Figure 4D, in green). Remarkably, this coincided with a transcriptional downregulation of *LjNSR1.1*, *NSR1.2*, *NSR2*, and *ENOD40-2*, together with a strong induction of *ENOD40-1* (Figure 2D), suggesting that the NSR-containing machinery modulating AS underwent a drastic rearrangement, potentially shaping the transcriptomic profile of the mature nodule. Therefore, the RNA processing program triggered by the inoculation with rhizobium did not remain constant throughout nodulation. Alternatively, the relative accumulation of mRNA isoforms dynamically evolved during nodule development, constituting a characteristic transcriptomic profile for each developmental stage.

## 4. Discussion

NSR proteins were initially denominated as MtRBP1 (for *Medicago truncatula* RNA Binding Protein 1; here MtNSR1) due to their ability to bind to RNAs in *M. truncatula* [8]. However, considering the large number of RBPs present in plants, we renamed the two closely related *A. thaliana* RBPs as NSRa and NSRb because their localization in nuclear speckles was consistent between the two species. Here, we report a comprehensive evolutionary analysis of the plant NSR proteins showing that, compared to other families of RBPs [26], NSRs constitute a rather small family of proteins. We propose that the origin of the NSR family predates the emergence of vascular plants, which appeared in the late Ordovician–Silurian [27], based on the presence of NSRs proteins in *M. polymorpha*, one of the few Bryophytes species sequenced. There is the presence of NSR proteins in all plant species since *M. polymorpha* suggests that they participate in key adaptive processes throughout evolution. In addition, the phylogenetic analysis indicates that the NSR family appeared in land plants, as indicated by the absence of NSR sequences in Algae. Notably, for the Bryophytes, all the species incorporated in the phylogenetic tree have at least one NSR protein hinting at the importance of their role in plant function and evolution. Considering the impact of gene (and genome) duplication in flowering plants in the expansion and diversification of gene families, where 65% of annotated genes have a duplicated copy [28], one of the major outcomes of the evolutionary analysis is the reductive trend for *NSR* genes faced by eudicots, where most of the species studied have only two or even only one *NSR* gene. Given that gene duplications are prevalent in the evolution of gene families in angiosperms, the NSR family shows an unexpected pattern in this plant group. Other RBP protein families containing three RRMs show] a different evolutionary history, with a high number of members accompanying gene duplications, being 24 in Arabidopsis, 19 in rice, and 37 in Poplar [26], in contrast to the tendency observed in NSR family.

The first studied member of NSR proteins was MtNSR1 due to its ability to bind the lncRNA *ENOD40* in *Medicago truncatula* [8]. Through phylogenetic inference, we found an additional gene encoding an NSR protein in this species, so far uncharacterized. Although the presence of conserved motifs in both proteins suggests that they might be involved in similar molecular mechanisms, their distinct transcriptional accumulation throughout the mature nodule (Figure 3) hints at specific roles in particular cell types. In the case of *L. japonicus*, the three NSR mRNAs (resulting from *NSR1* and the two isoforms of *NSR2*) exhibit differential transcriptional behavior during nodulation. Moreover, the two *LjENOD40* genes are also differentially regulated in nodule development, suggesting that alternative NSR-*ENOD40* modules may interact to fine-tune the mRNA populations at each developmental stage of nodule organogenesis. Considering that AtNSRa can recognize a plethora of lncRNAs in vivo [5], further research will be required to elucidate the NSR-lncRNAs network shaping the legume transcriptome governing symbiosis.

Some legume species have extra copies of NSR proteins compared with its closest relatives. Duplications found in *A. hypogaea* and *G. max* may be linked to the polyploid genomes of these important crop species. In the case of *A. hypogaea*, an allotetraploid derived from hybridization of *A. ipaensis* and *A. duranensis* [29], the expanded number of NSR proteins is probably the result of the retention of genes from its wild diploid parents. In the second case, the extra number of *NSR* genes could be due to two duplication events, followed by gene diversification, loss, and chromosome rearrangements that the *G. max* genome has undergone [30].

The phylogeny showed that *L. angustifolius* and *C. cajan* have two highly similar NSRs that clustered together respectively, prompting two alternative scenarios. The first possibility is that *NSR1* is a recent lineage-specific duplication and the member of *NSR2* was lost. An alternative hypothesis is that the *NSR2* gene accumulated many changes of residues due to selective forces, and for that reason, both sequences clustered as a recent lineage-specific duplication. We considered that the most plausible explanation would be the first scenario. Independent of the origin, our observations highlighted that for legumes, at least two NSR proteins were necessary. The difference between *L. angustifolius* and *C. cajan* is that the sequences of the first one belongs to the NSR1 clade, while for the latter species, both sequences are included in the NSR2 clade. This is relevant considering that until now, only NSR1 from the NSR1 clade has been characterized for binding *ENOD40*, and there is no information about the function of the NSR2 clade proteins in the family.

Besides the three conserved domains previously identified in NSRs from *Medicago* and other eudicot species [8], our analysis served to identify other conserved protein motifs and to reveal changes in the three previously identified domains that might have boosted functional diversification of NSR proteins.

Throughout evolution, NSR proteins faced a reductive trend within flowering plants where eudicots conserved only two genes coding for NSR proteins. Frequently, the evolutionary study of gene families provides evidence of gene number expansion through duplication that may result in neofunctionalization. In this sense, alternative splicing of *NSR* transcripts itself may contribute as an additional and important mechanism to promote molecular diversification conferring different abilities to the final proteins, and therefore distinct roles. In *A. thaliana*, a splicing variant of *NSRb* was identified [3]. We also found a splicing variant of one of the two *NSR* genes in the model legume *L. japonicus* (*LjNSR1.2*). Alternative splicing of *NSR* genes itself promotes diversification, potentially counteracting the reduction in the number of *NSR* genes in eudicots. Notably, *LjNSR1.2* still contains the C-terminal RRM domain responsible for RNA binding included in motifs 1 and 2, but has lost other conserved protein domains such as motif 6, which includes the NLS. Thus, although *LjNSR1.2* conserves the capacity to recognize RNAs, the lack of NLS, as well as others conserved motifs might prevent its participation in alternative splicing events, essentially nuclear. Accordingly, the lack of NLS suggests that sub-cellular localization of Embryophyte and Tracheophyte NSR may likely be essentially cytoplasmic, likely hindering their eventual participation in nuclear-localized alternative splicing. Further research will be needed to determine if Marchantia or Selaginella NSR proteins, as well as LjNSR1.2 or other NLS-lacking isoforms may enter the nuclei through an alternative mechanism.

The correlation between the transcriptional accumulation of *NSR* transcripts, *ENOD40s*, and the significant changes suffered by the population of alternatively spliced mRNAs throughout nodulation suggests a potential participation of NSR-*ENOD40* ribonucleoprotein complexes in AS modulation in this developmental context. Interestingly, *ENOD40* and *ASCO* are highly structured lncRNAs [3,31], although they exhibit no primary sequence conservation. The inherent evolutionary flexibility of lncRNAs hinders the identification of functionally related noncoding transcripts by simple sequence homology.

Our work sets the basis for the characterization of other members from the NSR family in different plant species and their lncRNA partners. Considering the wide range of mechanisms by which lncRNAs may modulate the action, localization, and stability of alternative splicing regulators [4], it would be of major importance to elucidate how *ENOD40*, *ASCO*, and yet undiscovered NSR-associated lncRNAs have co-evolved with their protein interactors to boost transcriptome and proteome diversity throughout evolution.

Recently, a phylogenomic survey of nitrogen-fixing root nodule (NFN) symbiotic plants revealed that, with the sole exception of *NODULE INCEPTION* (*NIN*), genes involved in this process are conserved in plants regardless of the loss of the symbiotic nodulation capacity of plant species [32]. In this sense, the presence of NSR proteins in nodulating and also in non-nodulating species is in agreement with the general trend reported. This raises the possibility that NSRs should participate in other developmental contexts different from symbiotic nodulation across land plants and that they may have been co-opted to orchestrate nitrogen fixation in Medicago and likely other NFN plant species within legumes and even across the other three angiosperm orders that develop NFN symbiosis.

## 5. Conclusions

Our work set the basis for the characterization of other members from the NSR family in different plant species and their lncRNA partners. Considering the wide range of mechanisms by which lncRNAs may modulate the action, localization, and stability of alternative splicing regulators [4], it would be of major importance to elucidate how *ENOD40*, *ASCO*, and yet undiscovered NSR-associated lncRNAs have co-evolved with their protein interactors to boost transcriptome and proteome diversity throughout evolution.

## Supplementary Materials

The following are available online at https://www.mdpi.com/2073-4425/11/2/207/s1, Figure S1: Phylogenetic analysis of the NSR protein family in plants, Figure S2: Protein sequence of *Medicago truncatula* RBP1/NSR1, Figure S3: Conserved motifs’ identification across NSR proteins, Table S1: List of species and protein annotations used for the phylogenetic analysis, Table S2: Expression dataset used for legumes. Table S3: List of DAS in *Medicago truncatula* and *Lotus japonicus* throughout nodule development, based on [17,18], using SUPPA [23].
